# MicroRNA profiling of the pig periaqueductal grey (PAG) region reveals candidates potentially related to sex-dependent differences

**DOI:** 10.1186/s13293-020-00343-2

**Published:** 2020-12-11

**Authors:** Klaudia Pawlina-Tyszko, Maria Oczkowicz, Artur Gurgul, Tomasz Szmatoła, Monika Bugno-Poniewierska

**Affiliations:** 1 Department of Animal Molecular Biology, National Research Institute of Animal Production, Krakowska 1, 32-083, Balice, Kraków, Poland; 2 Center for Experimental and Innovative Medicine, University of Agriculture in Kraków, Rędzina 1c, 30-248 Kraków, Poland; 3 Department of Animal Reproduction, Anatomy and Genomics, University of Agriculture in Kraków, al. Mickiewicza 24/28, 30-059 Kraków, Poland

**Keywords:** microRNAs, PAG, Brain, NGS, Pig

## Abstract

**Background:**

MicroRNAs indirectly orchestrate myriads of essential biological processes. A wide diversity of miRNAs of the neurodevelopmental importance characterizes the brain tissue, which, however, exhibits region-specific miRNA profile differences. One of the most conservative regions of the brain is periaqueductal grey (PAG) playing vital roles in significant functions of this organ, also those observed to be sex-influenced. The domestic pig is an important livestock species but is also believed to be an excellent human model. This is of particular importance for neurological research because of the similarity of pig and human brains as well as difficult access to human samples. However, the pig PAG profile has not been characterized so far. Moreover, molecular bases of sex differences connected with brain functioning, including miRNA expression profiles, have not been fully deciphered yet.

**Methods:**

Thus, in this study, we applied next-generation sequencing to characterize pig PAG expressed microRNAs. Furthermore, we performed differential expression analysis between females and males to identify changes of the miRNA profile and reveal candidates underlying sex-related differences.

**Results:**

As a result, known brain-enriched, and new miRNAs which will expand the available profile, were identified. The downstream analysis revealed 38 miRNAs being differentially expressed (DE) between female and male samples. Subsequent pathway analysis showed that they enrich processes vital for neuron growth and functioning, such as long-term depression and axon guidance. Among the identified sex-influenced miRNAs were also those associated with the PAG physiology and diseases related to this region.

**Conclusions:**

The obtained results broaden the knowledge on the porcine PAG miRNAome, along with its dynamism reflected in different isomiR signatures. Moreover, they indicate possible mechanisms associated with sex-influenced differences mediated via miRNAs in the PAG functioning. They also provide candidate miRNAs for further research concerning, i.e., sex-related bases of physiological and pathological processes occurring in the nervous system.

**Graphical abstract:**

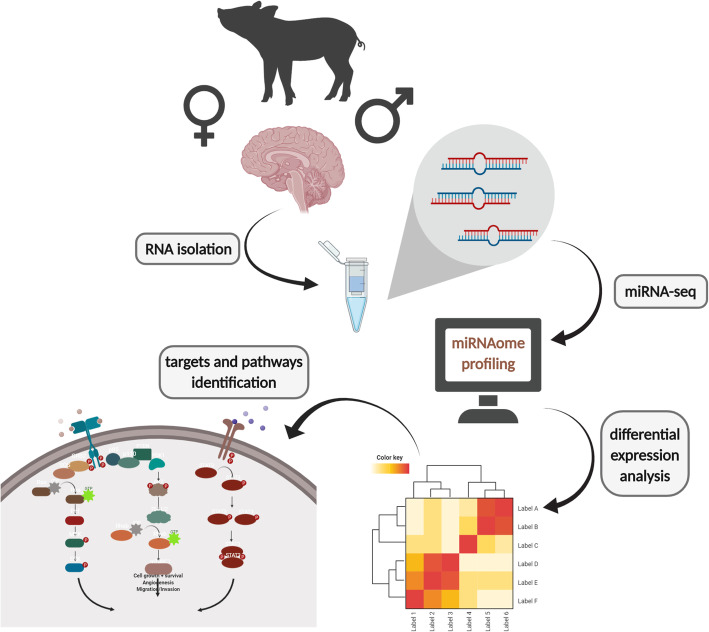

**Supplementary Information:**

The online version contains supplementary material available at 10.1186/s13293-020-00343-2.

## Background

The domestic pig (*Sus scrofa*) is an important animal not only for livestock production but also from the biomedical point of view as an alternate, large mammal model organism for the human [1–3]. This especially applies to neurological research because of the similarity of brain development (the growth pattern and the extent of peak brain growth at the time of birth), anatomy (i.e., gyral pattern and distribution of gray and white matter), and size between pigs and humans [4]. Thus, the pig is considered to be of great potential for broadening the knowledge of general neuronal and behavioral processes, as a subject of central nervous system (CNS) research, including neuroanatomy, neurobiology, and cognitive neuroscience [4]. It is also postulated for validation as an animal model for neurological and neuropsychiatric diseases such as schizophrenia and Alzheimer’s disease. So far, it has been used in imaging studies as an experimental model of traumatic brain injury, Parkinson’s disease and stroke, as well as to investigate serotonin and dopamine systems [4].

The brain is the central organ of the nervous system which is composed of many specialized structures and regions. The periaqueductal gray matter (PAG) is one of the mostly evolutionary conserved components of the brain. It is the central gray matter of the midbrain, to a large extent analogous to the gray matter of the spinal cord. It modulates various important functions including autonomic [5, 6], behavioral [7], pain [8], as well as defensive, reproductive, and maternal behavior [9, 10]. Moreover, it may be affected in many disorders, such as migraine [11], Wernicke’s encephalopathy [12], multiple sclerosis (MS) [13], and stroke [14, 15].

The brain, as a complex body organ, is characterized by a large diversity of miRNAs [16]. MicroRNAs are highly conserved, short (~ 21-23 nt) non-coding RNAs which orchestrate gene expression at the posttranscriptional level by binding to their targets—mRNAs [17]. miRNAs have been implicated to play crucial roles in most biological processes, not only in healthy tissues [18–21] but also in those undergoing pathological changes such as neoplastic transformation [22–24]. Therefore, microRNAs are also successfully used as biomarkers for diagnosis and prognosis [25–28]. They have also been identified to be engaged in the regulation of vital neuronal processes during neurogenesis and neuron functioning [29], as well as brain development, which is shown to undergo intensive changes of miRNA expression [30, 31].

To date, the miRNA expression profile of the pig brain tissue, namely cortex and cerebellum, has been identified by Podolska and colleagues [32].Nevertheless, brain regions are established to differ in gene expression [33] and miRNA profiles [34], and some of them can exert area-specific functions [34]. Moreover, the pig, with its 457 mature miRNA sequences deposited in miRBase Release 22.1, still stays behind the human (2654 mature miRNAs). Thus, in this study, we attempted to comprehensively characterize the miRNAome profile of the porcine PAG and, at the same time, broaden the whole pig miRNA profile and PAG profile. To this end, we applied next-generation sequencing, which allows for the detection of novel miRNA sequences as well as isomiR variants.

Additionally, the PAG region has been reported to play roles in many different neurodiseases and brain functions which are known to be sex-influenced, such as maternal behavior, pain [35, 36], and stroke [37]. Hence, we hypothesized that the PAG-expressed miRNAs may exhibit different expression profiles between sexes and, as a result, may be involved in sex-related differences. Therefore, we identified microRNAs differentially expressed between male and female samples, and biological pathways which they regulate, to pinpoint possible miRNA-involved mechanisms underlying sex differences and elucidate miRNA potential engagement.

Summing up, taking into account the important role that the PAG plays in the functioning of the brain, the aim of our work was to characterize its miRNA profile, including the identification of new miRNAs expressed in this region, as well as shed some light on the potential roles of miRNAs in shaping sex specific differences. It will not only broaden our knowledge on miRNAs and their significance in mechanisms occurring in the PAG, also those sex-related, but also provide data for further interspecies comparative studies, especially in humans, since the availability of brain samples is limited.

## Methods

### Research material

Periaqeductal grey samples were collected from 21 pigs maintained and slaughtered at 100 kg of weight at the Pig Testing Station of the National Research Institute of Animal Production in Pawłowice under the same housing and feeding conditions. The samples were frozen in liquid nitrogen immediately after collection, and stored at − 80 °C until RNA isolation. The animals (6 males and 15 females) belonged to the Polish 990 synthetic line of pigs, which is a hybrid of several breeds (Large White, Belgium Landrace, Duroc, German Landrace, Walsh Landrace, and Hampshire). The performed research did not require the approval of Animal Ethics Committee since meat from slaughtered animals is standard intended for consumption.

### MicroRNA sequencing

Total RNA extracted with the use of Direct-zol RNA Mini Prep kit (Zymo Research) according to the protocol was further subjected to the quantity and quality controls using a NanoDrop 2000 spectrophotometer (Thermo Fisher Scientific), and a TapeStation 2200 instrument (Agilent), respectively. NEBNext Multiplex Small RNA Library Prep Set for Illumina (New England Biolabs) was used to prepare miRNA libraries. This protocol starts with the 3′ adaptor ligation, followed by hybridization with the Reverse Transcription Primer and ligation with the 5′ adaptor. Obtained products were reverse transcribed, and PCR amplified, including 12 different indexed primers to allow multiplexing of the samples. The libraries were then subjected to size-selection with Novex 6% TBE PAGE gel (Invitrogen) electrophoresis, followed by ethanol (POCH) purification and precipitation. The concentration of the obtained libraries was measured with a Qubit 2.0 Fluorometer (Thermo Fisher Scientific), while a 2200 TapeStation instrument (Agilent) was used to assess their size. The libraries mixed with the PhiX control library (Illumina) were clustered on an Illumina Flowcell_v3 in a cBot cluster station and then sequenced on HiScan SQ (Illumina) system according to the manufacturer protocol.

### Bioinformatics analysis

The obtained raw reads were subjected to the following processing: conversion to FastQ files, demultiplexing with the use of the bcl2fastq software (Illumina), and quality control using the FastQC software [38]. Then, the obtained sequences were analyzed using UEA sRNA Workbench V4.6 [39] to identify known and potentially novel miRNA sequences. First, 3′ adaptor sequences were trimmed off and tRNA and rRNA sequences were discarded from the data. miRNA identification with the miRCat tool was performed with the default animal parameters except for minimum abundance (6 reads), minimum length (17 nt), and maximum length (25 nt) [40]. The identification was performed on the basis of the *Sus scrofa* genome (assembly Sscrofa 10.2) and miRBase v22.1 [41, 42]. Predicted candidate microRNA precursors were searched in the RNAcentral database v14 [43] to exclude those belonging to other non-coding RNA species. The remaining miRNA sequences were subjected to the identification of isomiRs that is microRNA length and sequence variants, using the isomiR-SEA software [44] and the default settings. Finally, detected miRNAs were analyzed using the DESeq2 software [45] to identify those differentially expressed between females and males. Since resultant miRNAs have false discovery rate (FDR) > 0.05, we chose statistically significant microRNAs differentially expressed at nominal *p* value ≤ 0.01 for further analyses. The most significant (*p* value ≤ 0.005) miRNAs were visualized with the pheatmap v1.0.12 package [46] using the R package v3.6.1 [47].

### miRNA-target gene interaction networks and enriched biological pathways

Interaction networks of the detected differentially expressed miRNAs and their database-deposited target genes were illustrated with the use of the miRNet 2.0 online platform [48, 49]. To this end, we chose “miRNAs” from the available options. The analysis was carried out with the default settings, except for a filter “Degree cutoff” which was set to 4.0, in order to increase the legibility of obtained networks and visualize the most important interactions.

The mirPath v.3 DIANA Tools web application [50] was used to determine biological processes enriched by the identified differentially expressed microRNAs (DE miRNAs) (females vs. males). The analysis was carried out employing experimentally validated target genes deposited in TarBase v7.0, as well as KEGG Pathway Database and Gene Ontology (GO) as reference databases.

### qPCR validation

Eleven microRNAs were chosen for the validation with the use of reverse transcription quantitative polymerase chain reaction (RT-qPCR) method. TaqMan Advanced miRNA cDNA Synthesis Kit (Thermo Fisher Scientific) was used to perform reverse transcription, while TaqMan Fast Advanced Master Mix (Thermo Fisher Scientific) and commercially available TaqMan microRNA Advanced Assays (Thermo Fisher Scientific) to run qPCR reactions in triplicates including non-template control (NTC) for each microRNA assay. qPCR reaction mix for one sample contained 10 μl TaqMan Fast Advanced Master Mix (2X), 1 μl TaqMan Advanced miRNA Assay (20×), 4 μl RNase-free water, and 5 μl diluted (1:10) cDNA template. All reactions were carried out according to the standard protocols on QuantStudio 7 Flex Real-Time PCR System (Thermo Fisher Scientific). miRNAs with the most stable expression profiles as specified by the NormFinder software [51] were selected as reference controls (miR-100-5p, miR-499a-5p). Relative expression levels were computed applying ΔΔCt method including reaction efficiency E [52] calculated with the use of the standard curve method.

## Results

### miRNAome profile of the PAG region

We conducted next-generation sequencing to characterize the miRNAome profile of the pig PAG area of the brain. As a result, from 3,567,080 to 6,033,979 raw sequences in individual samples were obtained. Subsequent filtering resulted in on average 3,311,434 sequences, which were further mapped to the *Sus scrofa* genome and miRBase 22.1. This allowed the identification of 237 unique known microRNAs, including 53 microRNAs* from the other strand, and 286 potentially new miRNA sequences (Supplementary File 1). The most predominantly expressed miRNAs were 22nt long. The miRNA profiling results were submitted to the NCBI GEO database and the following GEO accession number was assigned: GSE148302.

### Females vs. males differentially expressed microRNAs

**Fig. 1:**
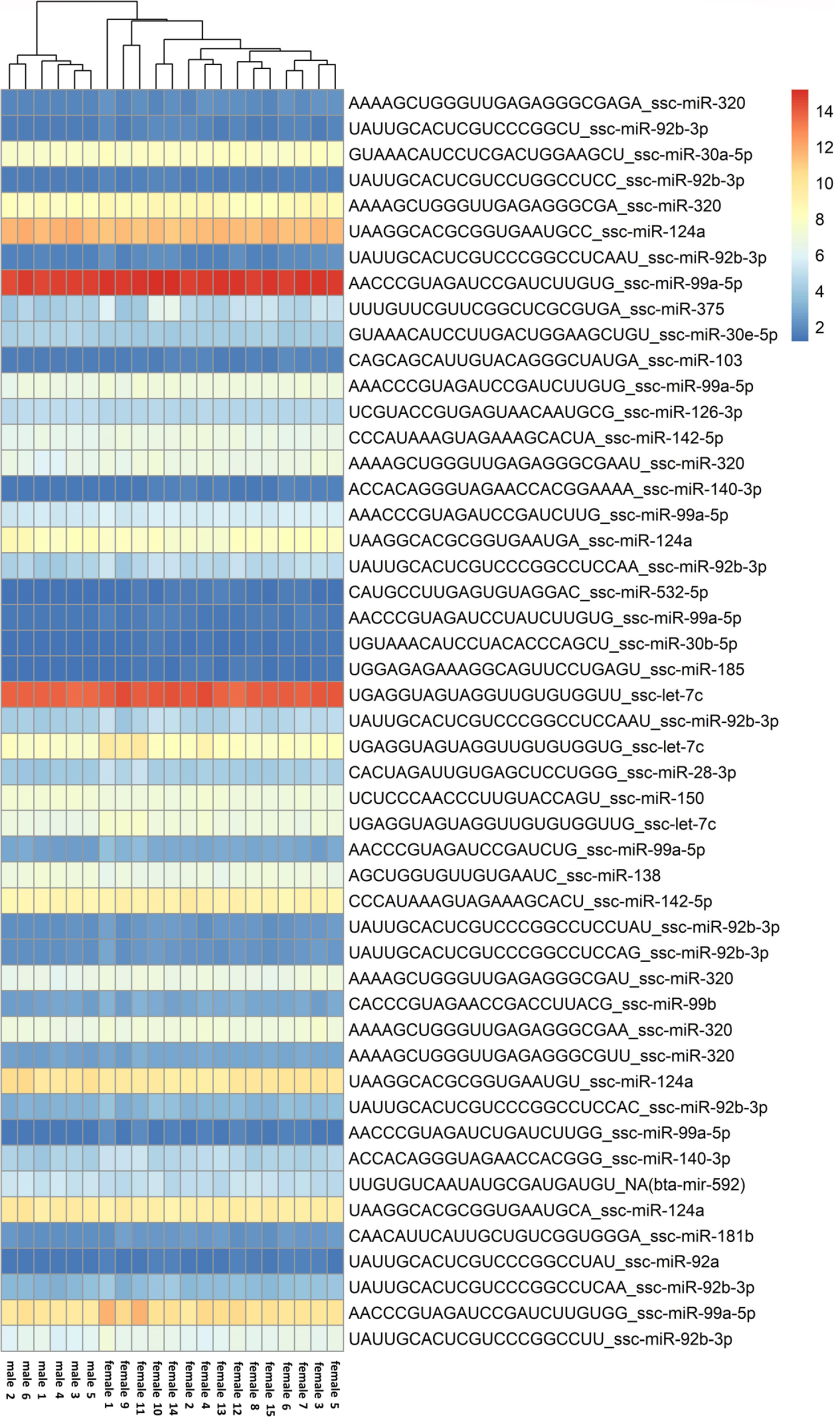
The expression pattern Heat Map. The Heat Map depicts the most significant (*p* value ≤ 0.005) differentially expressed miRNAs and their isomiRs (females vs males; males constitute the reference group) (R Package pheatmap)

Next, we performed differential expression analysis using the DESeq2 algorithm, which revealed 38 statistically significantly differentially expressed microRNAs (*p* value ≤ 0.01) in the female samples with reference to the male samples. Among them, 12 miRNAs were underexpressed and consisted of 29 isomiRs, whereas 26 microRNAs were overexpressed and included 84 isomiRs. All DE isomiR sequences originating from one microRNA showed the same direction of expression changes. The number of DE isomiRs belonging to one microRNA ranged from one (e.g., ssc-miR-150) to 17 isomiRs for ssc-miR-92b-3p. DE analysis details are present in Supplementary File 2, and the most significant (*p* value ≤ 0.005) differentially expressed microRNAs and their isomiRs are illustrated in Fig. 1.

### Pathways enriched by sex differentially expressed microRNAs

**Fig. 2:**
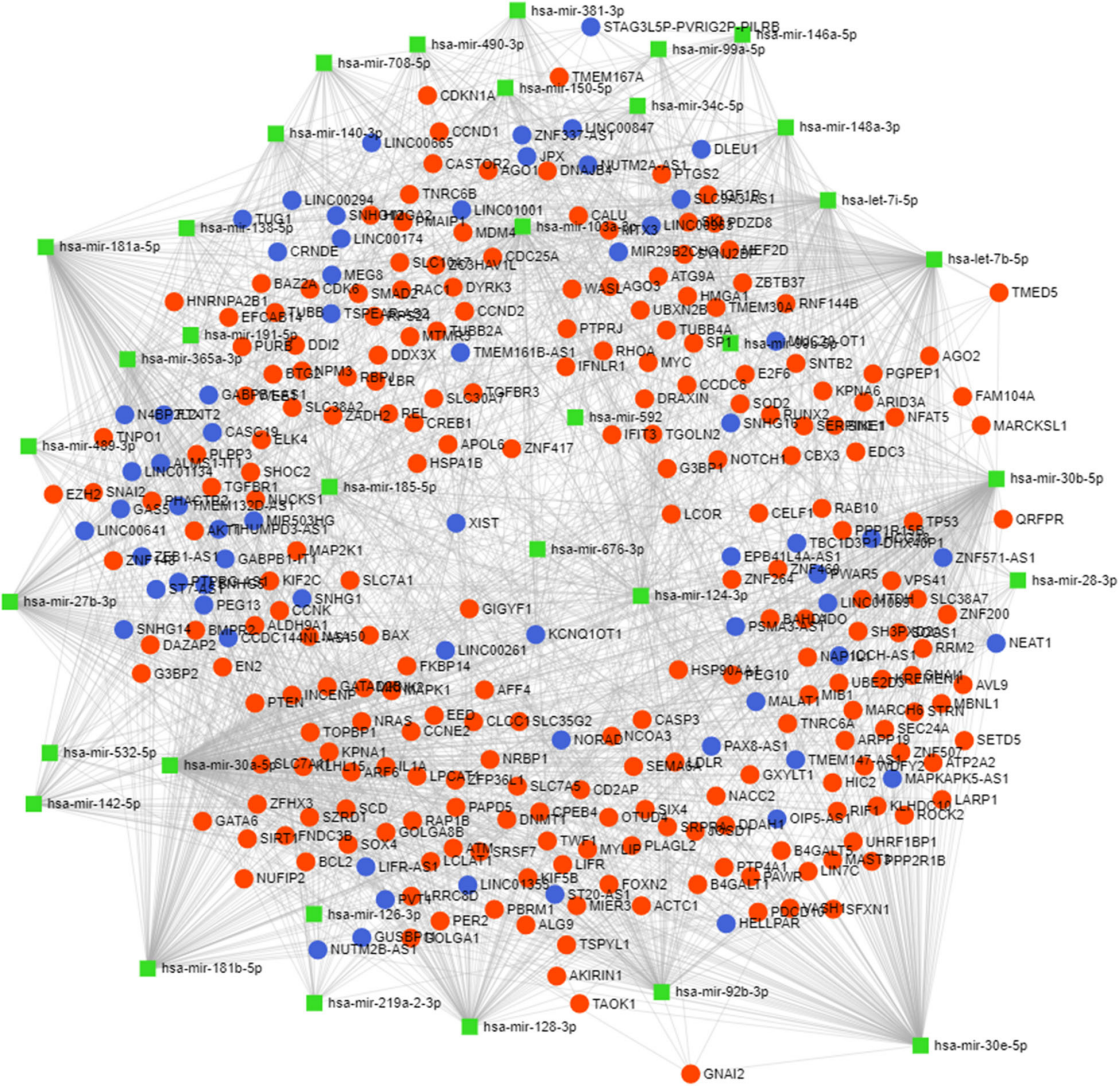
MiRNA interaction network. Global interaction network between the identified differentially expressed miRNAs (females vs. males), and predicted genes as well as long non-coding RNAs, which they target (miRNet web application). Green squares stand for DE microRNAs, red circles denote target genes, while blue circles stand for lncRNAs

**Fig. 3:**
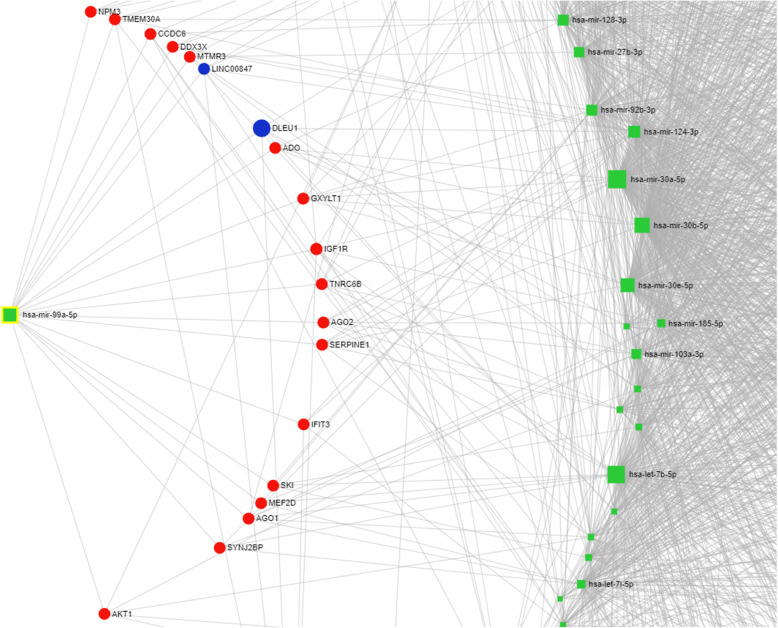
miR-99a-5p interaction network. Interaction network of differentially expressed (females vs. males) miR-99a-5p, and its predicted target genes as well as long non-coding RNAs (miRNet web application). Green squares denote DE microRNAs, red circles stand for target genes, and blue circles stand for lncRNAs

The visualization of interaction networks of DE microRNAs and their target genes is presented in Figs. *2* and *3*. The network, after degree filtering, embraces vast numbers of target genes (230) and long non-coding RNAs (66). It includes the following genes: ZNF148, MAP2K1, IGFR1, SERPINE1, TMEM30A, IL1A, NOTCH1, FOXN2, and many others. Whereas lncRNAs are represented by XIST, DLEU1, KCNQ1OT1, NEAT1, HELLPAR, and MALAT1.

**Fig. 4:**
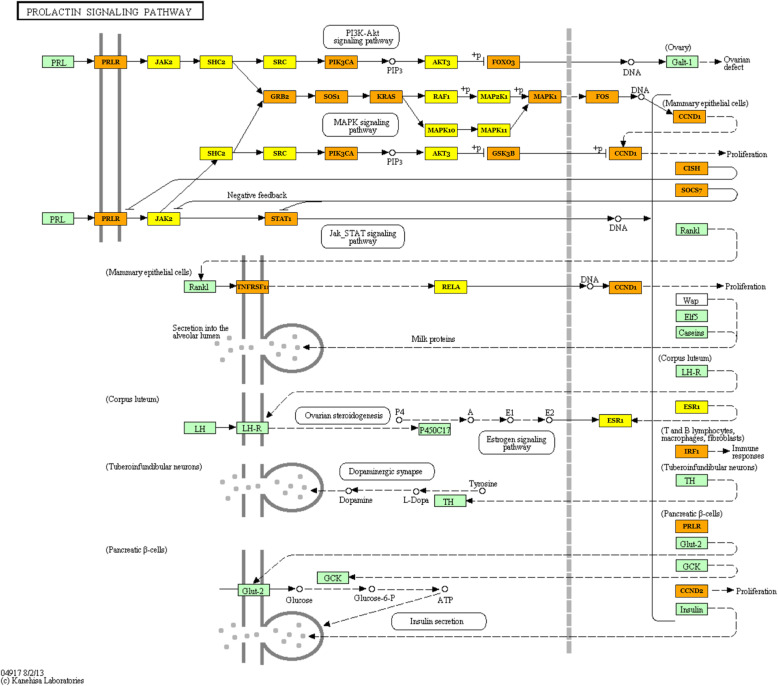
Prolactin signaling pathway (KEGG ID: hsa04917). A pathway identified as one of the most significantly enriched by the detected differentially expressed miRNAs in females in comparison to males. Green frame denotes genes engaged in the pathway. Genes targeted by identified DE miRNAs are marked yellow (genes included in one pathway) and orange (genes included in more than one pathway). Black arrows denote a molecular interaction or relation, whereas dotted arrows stand for an indirect link or unknown reaction

**Fig. 5:**
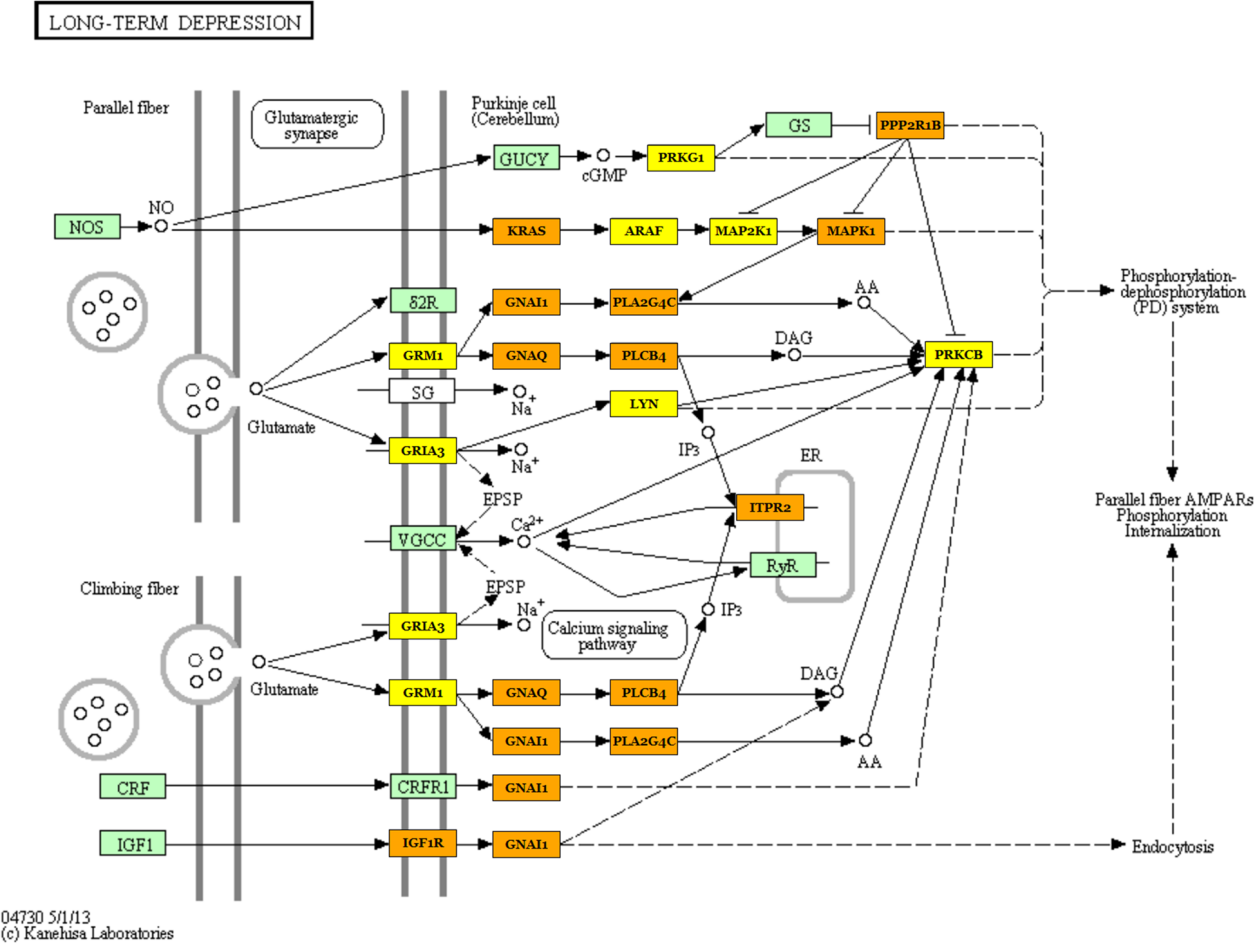
Long-term depression (KEGG ID: hsa04730). One of the most significantly enriched pathways by the detected differentially expressed between females and males microRNAs. Green frame stands for genes engaged in the pathway. Genes targeted by detected DE microRNAs are marked yellow (genes included in one pathway) and orange (genes included in more than one pathway). Black arrows stand for a molecular interaction or relation, while dotted arrows stand for an indirect link or unknown reaction

**Fig. 6:**
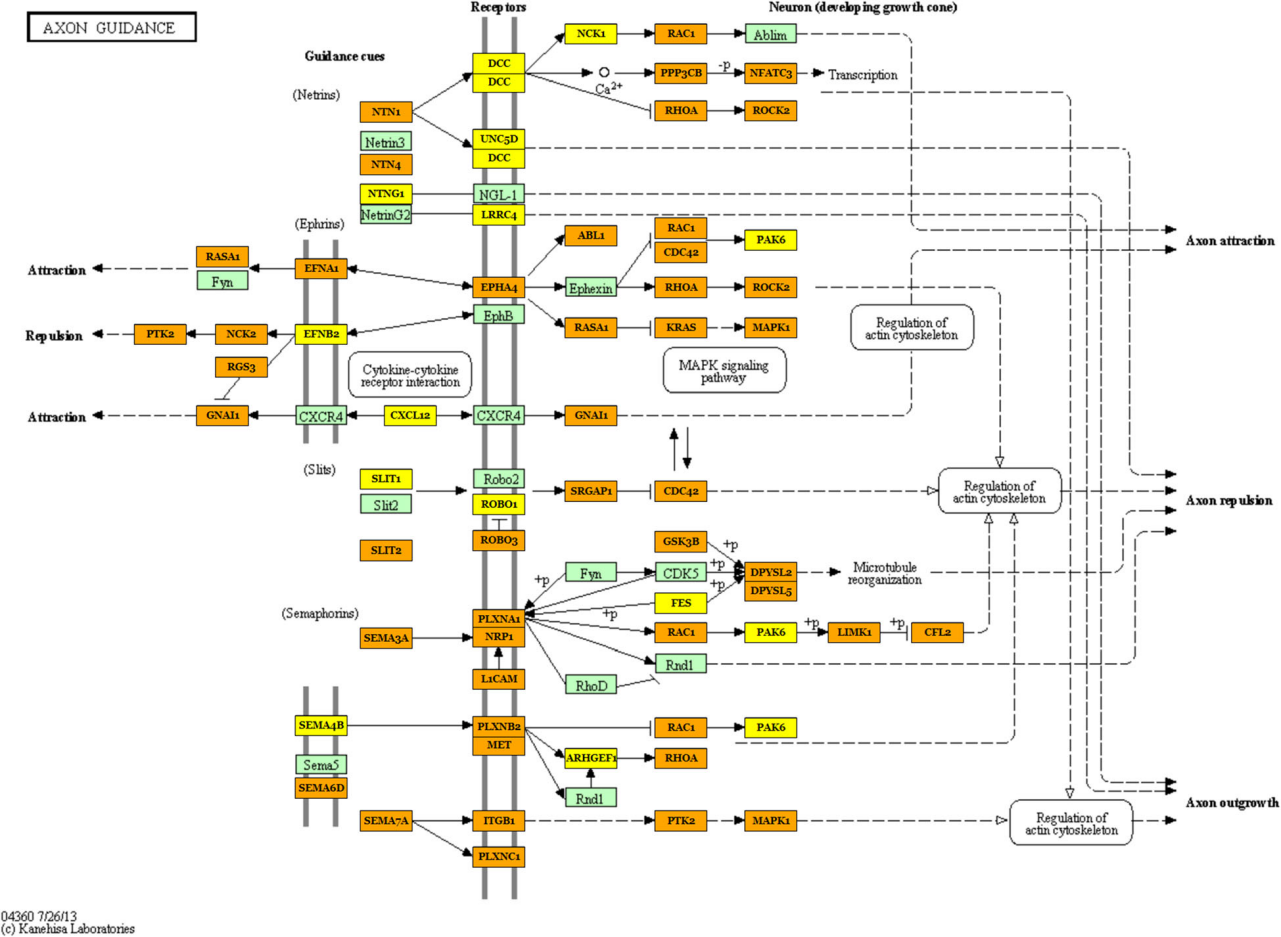
Axon guidance (KEGG ID: hsa04360). One of the most important pathways, enriched by the identified differentially expressed miRNAs in females vs. males. Green frame stands for genes involved in the pathway. Genes targeted by identified DE microRNAs are marked yellow (genes included in one pathway) and orange (genes included in more than one pathway). Black arrows denote a molecular interaction or relation, while dotted arrows denote an indirect link or unknown reaction

**Fig. 7:**
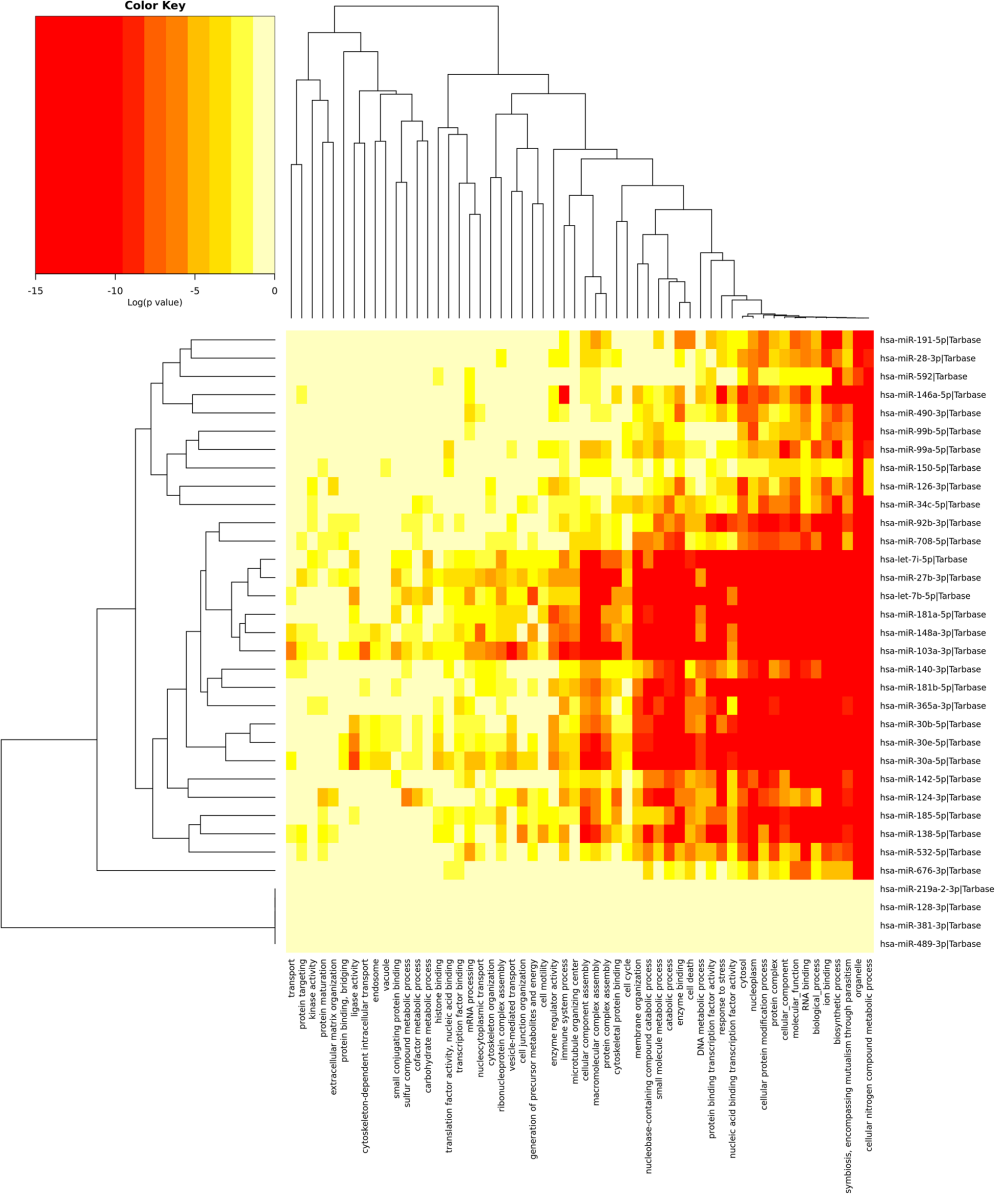
Heat map of significant GO terms enriched by the DE microRNAs (females vs. males) (DIANA-miRPath v3.0)

Moreover, we analyzed identified differentially expressed microRNAs (females vs. males) to elucidate their functions in the pig PAG region, with particular emphasis on a potential sex impact. As a result, numerous enriched KEGG pathways (Supplementary File 3) and GO terms (Supplementary File 4) were identified, encompassing a variety of biological processes. The most interesting over-represented KEGG pathways were fatty acid metabolism (hsa01212), estrogen signaling pathway (hsa04915), oocyte meiosis (hsa04114), endometrial cancer (hsa05213), progesterone-mediated oocyte maturation (hsa04914), prolactin signaling pathway (hsa04917) (Fig. *4*), long-term depression (hsa04730) (Fig. *5*), steroid biosynthesis (hsa00100), and axon guidance (hsa04360) (Fig. *6*) (Table 1). The enriched significant GO terms included response to stress (GO:0006950), enzyme regulator activity (GO:0030234), immune system process (GO:0002376), catabolic process (GO:0009056), generation of precursor metabolites and energy (GO:0006091), and transcription factor binding (GO:0008134) (Table 2). All identified GO terms and engaged miRNAs are shown in Fig. *7*.

**Table 1:** Significant KEGG pathways enriched in the identified miRNAs differentially expressed in females vs. males

KEGG pathway	Number of genes	Examples of genes	Number of miRNAs	Examples of miRNAs	***p*** value
**Fatty acid metabolism (hsa01212)**	34	*ACSL5*, *FASN*, *MCAT*, *ACADSB*, *ACSL3*, *PTPLB*, *ACOX1*, *PECR*, *ACOX3*, *HADH;*	19	miR-124-3p, **miR**-**142**-**5p**, **miR**-**27b**-**3p**, **miR**-**30a**-**5p**, **miR**-**181a**-**5p**;	6.23e-08
**Estrogen signaling pathway (hsa04915)**	73	*ESR1*, *BABBR1*, *FOS*, *HBEGF*, *ADCY1*, *SOS2*, *ATF2*, *ADCY7*, *NRAS*, *MAP2K2;*	30	**miR**-**103a**-**3p**, **miR**-**148a**-**3p**, **miR**-**27b**-**3p**, **miR**-**28**-**3p**, **miR**-**532**-**5p**;	4.50e-06
**Oocyte meiosis (hsa04114)**	78	*SLK*, *ESPL1*, *PPP1CA*, *FBXO5*, *CAMK2D*, *PPP2R5E*, *SMC1A*, *YWHAH*, *ADCY1*, *CCNB1;*	30	**miR**-**92b**-**3p, miR**-**30a**-**5p**, miR-365a-3p, **let**-**7i**-**5p**, miR-708-5p;	0.00015
**Endometrial cancer (hsa05213)**	40	*BRAF*, *GSK3B*, *ERBB2*, *SOS2*, *NRAS*, *MAP2K2*, *APC*, *PIK3CB*, *TCF7L2*, *RAF1;*	27	miR-138-5p, **miR**-**181b**-**5p**, miR-126-3p, **miR**-**146a**-**5p, miR**-**676**-**3p**;	0.00108
**Progesterone**-**mediated oocyte maturation (hsa04914)**	65	*BRAF*, *ADCY1*, *CCNB1*, *PGR*, *CCNA7*, *BUB1*, *FZR1*, *GNAI3*, *ANAPC2*, *ARAF;*	29	miR-592, **let**-**7b**-**5p**, miR-124-3p, **miR**-**181a**-**5p**, miR-30e-5p;	0.00116
**Prolactin signaling pathway (hsa04917)**	52	*ESR1*, *PRLR*, *FOS*, *GSK3B*, *STAT3*, *NFKB1*, *SOCS4*, *SOS2*, *CISH*, *JAK2;*	28	miR-490-3p, **let**-**7i**-**5p**, miR-124-3p, **miR**-**191**-**5p**;	0.00221
**Long**-**term depression (hsa04730)**	39	*BRAF*, *PRKCA*, *GNA12*, *NRAS*, *GNAS*, *GNA13*, *PLA2G4F*, *GNAQ*, *ITPR3;*	29	miR-124-3p, **miR**-**92b**-**3p**, miR-365a-3p, miR-708-5p, **miR**-**181a**-**5p**;	0.00420
**Steroid biosynthesis (hsa00100)**	14	*SS5D*, *TM7SF2*, *MSMO1*, *DHCR24*, *CYP51A1*, *SOAT1*, *LSS*, *SQLE;*	21	**miR**-**30a**-**5p, miR**-**30b**-**5p**, miR-30e-5p, **miR**-**148a**-**3p**;	0.00709
**Axon guidance (hsa04360)**	78	*EFNB2*, *SEMA6A*, *PLXNA2*, *GSK3B*, *MET*, *ROCK1*, *L1CAM*, *FES*, *RHOA;*	30	miR-124-3p, **miR**-**148a**-**3p**, **miR**-**676**-**3p**, **miR**-**99b**-**5p**;	0.04447

Upregulated miRNAs are in bold

**Table 2:** Significant gene ontology terms enriched in the identified miRNAs differentially expressed in females vs. males

GO term	Number of genes	Examples of genes	Number of miRNAs	Examples of miRNAs	***p*** value
**Response to stress (GO:0006950)**	1191	*TERF2*, *POLR2B*, *VPS4A*, *VAPB*, *ARPC5*, *NDUFS2*, *B2M*, *TAOK3*, *E2F7*, *PELI1;*	27	**let**-**7b**-**5p**, miR-126-3p, miR-138-5p, **miR**-**140**-**3p**, **miR**-**142**-**5p**;	< 1e-325
**Enzyme regulator activity (GO:0030234)**	376	*ARHGAP1*, *TBC1D20*, *TAOK3*, *TRIB3*, *RGS17*, *PFN1*, *ANP32E*, *PRLR*, *PKIA;*	19	**miR**-**146a**-**5p, miR**-**148a**-**3p**, **miR**-**28**-**3p, miR**-**34c**-**5p, miR**-**92b**-**3p;**	< 1e-325
**Immune system process (GO:0002376)**	742	*ARPC5*, *B2M*, *PELI1*, *TRIB3*, *BRK1*, *IRS2*, *CCL2*, *ADAM9*, *ACTB;*	26	miR-126-3p, miR-138-5p, **miR**-**140**-**3p, miR**-**142**-**5p, miR**-**146a**-**5p**;	< 1e-325
**Catabolic process (GO:0009056)**	1146	*RNF41*, *VPS4A*, *ABCD4*, *ERLIN1*, *YTHDC2*, *PPP1CA*, *RAB2B*, *RPA1;*	26	**miR**-**142**-**5p, miR**-**146a**-**5p, miR**-**181a**-**5p, miR**-**181b**-**5p, miR**-**185**-**5p**;	< 1e-325
**Generation of precursor metabolites and energy (GO:0006091)**	163	*NDUFS2*, *PPP1CA*, *FASN*, *PRKCA*, *PYGL*, *PFKP*, *COX8A*, *ACOX1;*	14	**let**-**7b**-**5p, let**-**7i**-**5p, miR**-**181b**-**5p, miR**-**185**-**5p, miR**-**27b**-**3p;**	1.11e-16
**Transcription factor binding (GO:0008134)**	233	*FHL2*, *TRIP6*, *ACTB*, *NACA*, *TCF3*, *HIPK2*, *STAT3*, *HOXA7*, *PAX6;*	16	**miR**-**181b**-**5p, miR**-**27b**-**3p**, miR-30e-5p, **miR**-**532**-**5p;**	4.10e-15

Upregulated miRNAs are in bold

### qPCR validation

The validation of miRNA sequencing results showed a high and significant Spearman correlation between next-generation sequencing (NGS) and qPCR for most of the analyzed miRNAs (from 0.56 to 0.95) (Table 3). Correlation coefficients were not significant for miR-23a-3p, -103a-3p, -339-5p, but the direction of expression changes obtained by these two methods was the same.

**Table 3:** The correlation coefficient of RNA-seq and qRT-PCR data

miRNA	R
miR-7-5p	0.95^***^
miR-21-5p	0.88^***^
miR-378a-3p	0.56^**^
miR-20a-5p	0.62^**^
miR-23a-3p	0.36^ns^
miR-103a-3p	0.21^ns^
miR-127-3p	0.78^***^
miR-210-3p	0.77^***^
miR-339-5p	0.12^ns^

*R* Spearman correlation coefficient with *p* value (***p < 0.001; **p < 0.01; *ns* not significant)

## Discussion

MicroRNAs have emerged as key regulators of a plethora of biological processes, including those occurring in the central nervous system. Accumulating evidence on their vital roles in different aspects of neurodevelopment and functioning provides resources to elucidate the physiology and pathophysiology of this complex system. The implementation of high throughput technologies allows further, deeper insight into the subject and facilitates characterization of this still unraveled and incomplete picture of miRNA repertoire and its regulatory influence.

### miRNA profiling reveals inter-species conservative nature and ubiquitous expression of ssc-miR-9, -26a, and -99a-5p in different brain regions

To date, an approach was made to identify miRNA-dependent regulatory mechanisms related to brain development ontogenesis in pigs. Using the microarray technology and qRT-PCR, Podolska and colleagues [32] determined miRNA expression levels in the developing pig brain. Noteworthy, almost half of the known miRNAs detected in our study overlaps with their results, which suggests that they may be pig, brain-ubiquitous miRNAs. The differences of miRNA profiles obtained by our research teams may stem from different methods applied (microarrays vs. NGS), developmental stages (fetuses and piglets vs. adult pigs), brain regions (cortex and cerebellum vs. PAG), and available at the time reference miRNAs (miRBase 15.0 vs. 22.1). Likewise, 18 miRNAs were common for this study and human brain miRNA profiling studies which established the vital roles of miRs in a variety of neuron-occurring processes, and in disruptions of neuronal functions [53]. This stays in agreement with the nature of the examined tissue and, as such, confirms the obtained miRNA profile results.

What is more, numerous isomiRs (sequence and length variants) were detected, also those differing in the 5′ end sequences comprising alternative seed sequences (Fig. 1, Supplementary File 2). This implies that they can undergo target gene switching, and, as a result, influence different biological pathways [54, 55], which further can have a major impact on the whole tissue functioning.

The most abundantly expressed in the examined in this study pig PAG samples were ssc-miR-9, -26a, and -99a-5p (Supplementary File 1). Of those, miR-9 is classified as a NeurimmiR that is a brain-specific microRNA involved in the mediation of the immune system functioning [56], while miR-26a and -99a are brain-enriched miRs [53, 57]. They were also identified by Podolska and colleagues [32] in the developing pig brain (cortex and cerebellum). These miRNAs were shown to play important roles in neuronal functioning in different species, such as memory, synaptic plasticity, and neuroinflammation [56, 58]. Their detection in the present study indicates their inter-species conservative nature and ubiquitous expression in different brain regions. However, their exact significance in the PAG physiology remains to be elucidated.

### Female vs. male differential expression analysis identifies candidate miRNAs associated with neurological and psychiatric disorders as well as pain modulation

The performed analysis of miRNAs differentially expressed in females in comparison to males revealed 38 microRNAs (Supplementary File 2), of which five (miR-146b-5p, -126-3p, -103a-3p, -181a-5p, -181b-5p) overlap with those detected by Meder and colleagues [59] in human peripheral blood as influenced by sex. Furthermore, Munoz-Culla and colleagues [60] profiled miRNAomes of peripheral blood leukocytes of patients with relapses and remission of multiple sclerosis, and revealed sex-dependent differences. Among miRNAs found as differentially expressed in female but not in male remission patients were miR-27b-3p, 30a-5p, -30e-5p, and -148a-3p, which were also established in this study as differentially expressed (Fig. 1, Supplementary File 2). It should be noted that our studies comprised different species, tissues, methodological approaches, sample numbers, and last but not least different miRBase releases, which may explain those few common miRNAs, which, however, are still of the importance because they may constitute the most conservative miRNAs and confirm the obtained results.

miR-99a-5p was not only determined in our study as abundantly expressed in the PAG but was also shown to be upregulated in the female samples in comparison to the male samples (Fig. 1, Supplementary File 2). Considering the fact that the expression of this miRNA is stroke-influenced, these results may suggest that this miR may be one of the elements of the regulatory network associated with different susceptibility of females and males to ischemic stroke [37]. Other miRNAs identified as sex differentially expressed in our study (miR-124, -148a, -let-7i, -320d, -320e, -30a, -126, -219) may also constitute components of this network because of the fact that they were determined as stroke-dependent microRNAs as well [61].

miR-99a-5p poses an interesting subject of further research especially since the miRNet miRNA-target interaction analysis (Figs. 2 and 3) showed that it might exert gene regulatory influence on a handful of central nervous system crucial genes. For example, high expression levels of DEAD-box helicase 3 X-linked (DDX3X) encoding adenosine triphosphate (ATP)-dependent RNA helicase are correlated with poor survival outcome in human gliomas [62]. Furthermore, insulin-like growth factor-1 receptor (IGF1R) was shown to take part in the regulation of cortical neuronal migration, axon formation and polarity of those neurons [63], and brain development in a region-specific manner [64]. Argonaute 1 (AGO1) and Argonaute 2 (AGO2) comprise another worth attention targets since they code for proteins essential for proper RNA-induced silencing complexes (RISC) assembly and function, and, as a result, determine global miRNA abundance. Of note, disrupted RISC assembly within CNS was observed during autoimmune demyelination [65]. Another gene, serpin family E member 1 (SERPINE1), was established as a key regulator in glioblastoma dispersal [66] and may constitute a promising therapeutic target in Alzheimer’s disease [67]. Whereas myocyte enhancer factor 2D (MEF2D) gene belongs to the MEF2 family of transcription factors, which was shown to play important roles during brain development and function. Moreover, these transcription factors were suggested to exert a complex and profound influence on memory formation [68]. The MEF2 family was also emphasized as a risk factor for neuronal developmental disorders, psychiatric disorders such as schizophrenia, and mental illnesses such as autism [69].

Interestingly, other identified herein DE miRNAs in females in comparison to males were also shown to have altered expression levels in psychiatric disorders pathogenesis, namely miR-219, -181b, -124, -320, -128, and-30a [70]. Notably, two research teams investigating changes of miRNA profiles in schizophrenia revealed numerous dysregulated miRNAs, of which miR-30b, -92, -30a-5p [71] and miR-128, -138-, 148a, -150, -27b, -28, -381, -489, -99a, -181a, -181b [72] were also found as differentially expressed (females vs. males) in our study (Fig. 1, Supplementary File 2). This seems to provide a valuable stimulus for further extended studies since the PAG was hypothesized to play roles in schizophrenia as a structure mediating basic emotions and primordial self-consciousness [73, 74]. A brief overview of differentially expressed miRNAs identified in this study and various brain diseases is presented in Table 4.

**Table 4:** Examples of miRNAs associated with neurological or neuropsychiatric diseases, identified as differentially expressed in our study

miRNA name	Up or downregulated in females vs. males	Disease
miR-381-3p	−	Sc [72]
miR-34c	−	AD [75], PD [76]
miR-489	−	ASD [77], Sc [72]
miR-320	−	ASD [78], S [61]
miR-27b-3p	−	S [79], AD [80], MS [60], Sc [72]
miR-181a	−	Sc [72]
miR-181b	−	S [81], Sc [72]
miR-30e-5p	−	MS [60]
miR-30a-5p	−	MS [60], Sc [71]
miR-28-3p	−	Sc [72]
miR-30b-5p	−	MS [82], Sc [71]
miR-185	−	PD [83], MD [84]
miR-150	−	S [85], Sc [72]
miR-191	−	AD [86]
miR-124a	+	S [61]
miR-128	+	Sc [72]
miR-126-3p	+	S [61]
miR-92a	+	Sc [71]
miR-99a-5p	+	S [61], Sc [72]
let-7i-5p	+	S [61]
miR-138	+	S c[72]
miR-148a-3p	+	MS [60], S [61]
miR-219b-3p	+	S [61]

*Sc* schizophrenia, *AD* Alzheimer’s disease, *PD* Parkinson’s disease, *ASD* autism spectrum disorders, *MS* multiple sclerosis, *S* stroke, *MD* major depression

Additionally, schizophrenia is also characterized by sex-driven differences which are reflected in the age of onset, symptoms, and response to treatment [87–90]. The exact pathogenesis of these differences remains vague and may embrace a variety of genetic and environmental factors; nevertheless, identified herein sex differentially expressed miRNAs emerge as candidates for more extensive research, especially since they coincide with biological pathways relevant to neuron functioning, such as PI3K-Akt signaling pathway (Supplementary file 3).

One of the most intensively studied roles of the PAG is the modulation of pain [8, 91], which was established to differ substantially between males and females [35, 36]. Accumulating evidence suggests that this sex differential pain sensitivity stems from not only neuroanatomical features but also the influence of sex hormones, and the degree of proinflammatory immune response, which is in line with estrogen signaling pathway (Table 1) and immune system process (Table 2, Fig. 7) identified in this study as enriched by miRNAs differentially expressed between males and females.

Furthermore, numerous studies allowed characterization of miRNAome signatures of patients suffering from different types of pain and identification of miRNAs with potential to serve as pain subtype and intensity biomarkers [92–100]. A handful of those miRNAs was also identified in this study as differentially expressed in the PAG region between females and males (Supplementary file 2). These common miRNAs embrace miR-126-3p which was detected in chronic musculoskeletal pain [99], migraine [98], and complex regional pain syndrome [92] as well as miR-320a identified in chronic musculoskeletal pain [99], complex regional pain syndrome [92], persistent axial musculoskeletal pain [96], and fibromyalgia suffering patients [93]. Moreover, Linnstaedt and colleagues suggested the existence of sex influence in the case of miR-320a [96]. We also detected miR-181a and -142-5p which were differentially expressed in complex regional pain syndrome [92] and migraine [94, 95], while miR-150-5p was reported in chronic musculoskeletal pain [99]. Two more miRNAs identified in complex regional pain syndrome (let-7c, miR-185) [92] and fibromyalgia suffering patients (miR-103a-3p, miR-30b-5p) [93] were also DE in this study. Furthermore, Leinders and colleagues reported miR-146a-5p to be altered in peripheral neuropathies [97], while Tavares-Ferreira’s team identified altered expression of miR-138 in lingual nerve neuromas [100]. When it comes to profiled migraine miRNAs, the differential expression of miR-27b [94] and miR-34c, -124-3p, -375, and -532-5p [95] was also shown in our study in females vs. males.

These pain-associated miRNAs may constitute interesting subjects of additional research focused on deciphering the meaning and exact roles of microRNAs in sex-driven pain differences; especially since they were identified in this study as overrepresented in the aforementioned estrogen signaling pathway and immune system process (Supplementary file 2, 3). This implies they may underlie sex-related pain sensitivity through sex hormone and immune response-driven mechanisms; however, it requires further research. Moreover, miR-320a was the most commonly identified in different types of pain, which suggests it may be a universal pain mediator. It was also reported that it may undergo sex influence [96]. Altogether, the previous results along with our study indicate it may be an especially promising biomarker to be tested.

### Pathways enriched in miRNAs differentially expressed between females and males play roles in crucial neuronal processes

Among identified herein miRNA overrepresented pathways was PI3K-Akt signaling pathway (Supplementary file 3). This pathway was established to be of profound significance in emotional regulation, language, behaviors, and complex cognition, while disruptions of this multifaceted interaction network were regarded as a root cause of different neuronal diseases, such as epilepsy, autism, and schizophrenia [101]. Moreover, also the components of the axon guidance pathway (Fig. 6) are being associated with schizophrenia and other psychiatric disorders [102]. When it comes to the physiological role of this pathway, it is responsible for a nervous system peculiar feature, because it directs growing axons toward their targets, which creates the complex wiring of the neuronal tissue.

Another identified key neurodevelopmental pathway was long-term depression (Fig. 4, Table 1), which is a form of synaptic plasticity—a biological process thought to contribute to memory and learning. Growing evidence supports miRNA involvement in this mechanism [103], which is in line with our results. Moreover, this form of synaptic plasticity has been investigated in terms of its involvement in chronic pain [104, 105]. Since the PAG plays a key role in the modulation and perception of pain [8, 91], the engagement of this pathway in mechanisms underlying the PAG-associated pain is worth further investigation. What is more, long-term depression pathway-enriched miRNAs identified in this study may provide potential candidates for such research (Supplementary file 3).

Of note, other synapse-related KEGG pathways such as GABAergic synapse, glutamatergic synapse, dopaminergic synapse, and cholinergic synapse (Supplementary file 3) were identified in this study as enriched by the detected microRNAs. These pathways are shown to be involved in the transmission of signals to and from the PAG [91]. Furthermore, estrogen signaling pathway, oocyte meiosis pathway, and prolactin signaling pathway (Fig. 4, Table 1) were statistically significantly overrepresented by the identified DE miRNAs. The enrichment of these pathways is not surprising having taken into consideration the investigated influence of miRNA-related sex differences on biological processes. What is more, prolactin, which is responsible for maternal behavior, was established to induce the activity of the PAG region, which is one of nuclei of the sociosexual and maternal brain [106].

The identification of pathways crucial for nervous tissue biology confirms the role and significance of miRNAs for the PAG physiology, with a special emphasis on those potentially taking part in mechanisms responsible for sex differences.

## Conclusions

The comprehensive analysis of the miRNAome profile of the porcine PAG tissue enabled us to determine conservative miRNAs characteristic for the brain tissue, such as miR-9, -26a, and -99a-5p. Of note, this is the first study to reveal the repertoire of potentially novel sequences and isomiR signatures in the pig PAG region, which sheds some light on the multifaceted influence of miRNA expression on brain functioning and gives a stimulus for future research. Furthermore, the comparison of miRNAome profiles in terms of sex influence revealed numerous miRNAs with potential to underlie a range of processes and diseases manifested in sex-related manner. Further analysis allowed identification of biological pathways essential for neurodevelopment and neuronal functioning, as well as other molecular processes which may constitute a part of mechanisms driving sex differences. The identification of the present study miRNAs in human brain-focused studies may act as the confirmation of the obtained results, and, at the same time, as the confirmation of significant roles of miRNAs in the neurodevelopmental processes. Additional research is warranted to elucidate the exact relationships between the identified miRNAs and physiological processes being disrupted in the course of different diseases in humans in a sex-dependent manner, and, as a result, to investigate the clinical potential of those microRNAs for treatment.

## Perspectives and significance

Taking into consideration the problematic access to brain tissues of patients, animal models, especially the pig with its physiological resemblance to the human, constitute potent sources to carry out such research. Therefore, obtained in this study results provide potential miRNAs and pathways to be investigated in the future research embracing the higher number of samples and the species of particular interest that is the human, to gain wider and more comprehensive view of brain and sex-driven mechanisms,.

## Supplementary Information

**Additional file 1.** Supplementary File 1. Detailed data on the identified miRNAs. Table containing data about microRNAs detected in all investigated samples with the use of the UEA sRNA Workbench software. “NA” in the “miRNA name” column denotes potentially novel miRNAs identified in this study, which additionally are bolded.

**Additional file 2.** Supplementary File 2. Results of the differential expression analysis between females and males using the DESeq2 algorithm (p value≤0.05).“log2FoldChange” – the binary logarithm of the Fold Change parameter; “NA” in the miRNA name column stands for potentially novel microRNAs identified in this study.

**Additional file 3.** Supplementary File 3. List of KEGG pathways enriched by the detected differentially expressed in females microRNAs (p value≤0.05).

**Additional file 4.** Supplementary File 4. List of GO terms enriched by the identified differentially expressed in females microRNAs (p value≤0.05).

## Abbreviations

PAG: Periaqueductal grey
DE miRNAs: Differentially expressed microRNAs
miR: MicroRNA
FDR: False discovery rate
KEGG: Kyoto Encyclopedia of Genes and Genomes
GO: Gene ontology
RT-qPCR: Reverse transcription quantitative polymerase chain reaction
NTC: Non-template control
GEO database: Gene Expression Omnibus database
NGS: Next-generation sequencing
RISC: RNA-induced silencing complex
CNS: Central nervous system
DDX3X: DEAD-Box Helicase 3 X-Linked
ATP: Adenosine triphosphate
IGF1R: Insulin-like growth factor-1 receptor
AGO1: Argonaute1
AGO2: Argonaute2
SERPINE1: Serpin family E member 1
MEF2D: Myocyte enhancer factor 2D
PI3K-Akt: Phosphatidylinositol-3-kinase and protein kinase B
Sc: Schizophrenia
AD: Alzheimer’s disease
PD: Parkinson’s disease
ASD: Autism spectrum disorders
MS: Multiple Sclerosis
S: Stroke
MD: Major depression
GABA: Gamma-Aminobutyric acid
